# Improving the Tribological Properties of WE43 and WE54 Magnesium Alloys by Deep Cryogenic Treatment with Precipitation Hardening in Linear Reciprocating Motion

**DOI:** 10.3390/ma17092011

**Published:** 2024-04-25

**Authors:** Adrian Barylski, Krzysztof Aniołek, Grzegorz Dercz, Izabela Matuła, Sławomir Kaptacz, Jan Rak, Robert Paszkowski

**Affiliations:** Faculty of Science and Technology, Institute of Materials Engineering, University of Silesia in Katowice, 75 Pułku Piechoty Street 1A, 41-500 Chorzów, Poland; krzysztof.aniolek@us.edu.pl (K.A.); grzegorz.dercz@us.edu.pl (G.D.); izabela.matula@us.edu.pl (I.M.); slawomir.kaptacz@us.edu.pl (S.K.); jan.rak@us.edu.pl (J.R.); robert.paszkowski@us.edu.pl (R.P.)

**Keywords:** WE43 and WE54 magnesium alloys, XRD, linear reciprocating motion, friction, wear, morphology of wear track, morphology of wear products

## Abstract

This paper presents the results of tribological tests on WE43 and WE54 magnesium alloys with rare earth metals performed in linear reciprocating motion for four different material couples (AISI 316-L steel, silicon nitride—Si_3_N_4_, WC tungsten carbide, and zirconium dioxide—ZrO_2_). Additionally, magnesium alloys were subjected to a complex heat treatment consisting of precipitation hardening combined with a deep cryogenic treatment. The study presents the effect of deep cryogenic treatment combined with precipitation hardening on the tribological properties of WE43 and WE54 alloys. Tribological tests revealed the most advantageous results for the magnesium alloy—AISI 316-L steel friction node. For both alloys tested after heat treatment, a nearly 2-fold reduction in specific wear rate has been achieved. Furthermore, microscopic examinations of the wear track areas and wear products were performed, and the wear mechanisms and types of wear products occurring in linear reciprocating friction were determined. Wear measurements were taken using the 3D profilometric method and compared with the results obtained from calculations performed in accordance with ASTM G133 and ASTM D7755, which were modified to improve the accuracy of the calculation results (the number of measured profiles was increased from four to eight). Appropriately selected calculation methods allow for obtaining reliable tribological test results and enabling the verification of both the most advantageous heat treatment variant and material couple, which results in an increase in the durability of the tested alloys.

## 1. Introduction

Magnesium alloys with rare earth metals are gaining popularity in various industries due to their high strength and low density, which makes them ideal structural materials for applications where weight reduction is crucial. These alloys are widely used in the automotive, aerospace, and land transport industries. They contribute to reducing fuel consumption and improving the dynamic characteristics of vehicles. Magnesium alloys are also used in many other industries, including as materials for biomedical applications. Magnesium and magnesium alloys are characterized by excellent biocompatibility, good mechanical strength, and biodegradation properties. The modulus of elasticity of magnesium is approximately 45 GPa, which is of the same order of magnitude as that of bones (15–25 GPa), resulting in reduced stress shielding. The density of Mg alloys ranges from 1.74 to 1.84 g/cm^3^, depending on the alloy components, and is nearly equal to that of bone (1.8–2.1 g/cm^3^). Magnesium alloys with rare earth metals designed by the company (MAGNEZIX^®^-Syntellix AG, Hanover, Germany) were officially accepted as a temporary implant material in 2013 [1,2,3]. The degradation time in in vivo applications is longer than for the other Mg alloys. Implants can retain their mechanical properties so that the tissue has time to heal and the amount of degradation product released is reduced. They are marketed as bioabsorbable screws, pins, and wires. A major advantage of magnesium alloys is that their degradation products are non-toxic and beneficial for new bone formation. The main disadvantages of these alloys are low plasticity, poor corrosion resistance in water and alkaline environments (low standard electrode potential of −2.372 V), and poor resistance to tribological wear [3]. Rare earth metals (RE) are alloying elements that increase the mechanical strength, improve corrosion resistance, and increase the creep resistance of magnesium. Yttrium (Y), gadolinium (Gd), cerium (Ce), and neodymium (Nd) are the most commonly used rare earth elements for Mg-based alloys for biomedical applications [4,5,6,7,8]. The most advantageous mechanical properties are obtained when Zn and/or Zr are combined with rare earth elements, which can be found, among others, in WE43 and WE54 commercial alloys [9,10].

This paper proposes the application of a complex heat treatment in the form of precipitation hardening combined with deep cryogenic treatment (DCT) with appropriately selected solution treatment and aging temperatures depending on the Y content of the alloy composition, investigates tribological properties, determines wear mechanisms, and identifies wear products. Tests were carried out in various material couples (WE43, WE54—AISI 316-L, Si_3_N_4_, WC, and ZrO_2_ alloys). The present study contributes to the state of knowledge in both the subject of magnesium alloys with rare-earth metals and the tribological studies themselves in linear reciprocating motion described by the research team of A. Zabala et al. [11]. In this article, we suggest that the recommendations of ASTM G133 and ASTM D7755 [12,13] should be modified regarding the measurement of the average wear track area.

## 2. Materials and Research Methodology

The research object consisted of WE43 and WE54 magnesium alloys, manufactured by Luxfer MEL Technologies (Manchester, UK). Research materials were supplied in the form of rods, ∅ = 25.4 mm in diameter (1 inch). For tribological tests, specimens with the nominal diameter of the rod and a height of 5 mm were prepared. The chemical composition according to the manufacturer’s certification is presented in Table 1.

The process of precipitation hardening was conducted in the FCF-5M laboratory muffle furnace (Czylok, Jastrzębie-Zdrój, Poland) in the air atmosphere. The temperature of solution treatment, aging, and heat treatment have been determined in previous studies [14,15,16]. For both alloys, the solution treatment (Sol) temperature was 545 °C and the solution treatment time was 8 h. The aging (Age) temperature for WE43 alloy was 225 °C and 250 °C for WE54 alloy. The aging time was 24 h. The precipitation hardening process has been combined with a deep cryogenic treatment (DCT) carried out over 24 h at liquid nitrogen temperature (−196 °C)—before and after the aging process. This yielded 10 different variants of test material. Table 2 presents a summary of the treatment carried out, together with a detailed description of the designations for all variants.

**Figure 1:**
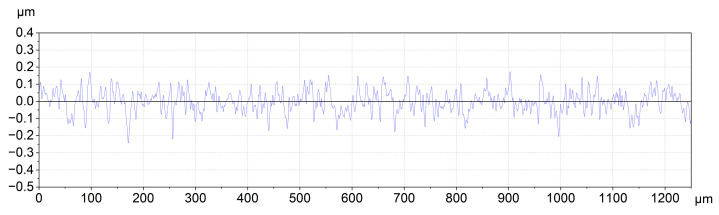
An example of surface roughness measurement of WE43 magnesium alloy measured with a Surftest SJ-500 contact profilometer.

Grinding of the specimens was carried out according to an in-house procedure using papers of grit size 320–4000 in order to achieve a uniform surface roughness of Ra = 0.05 μm. The roughness of the specimens was measured with the Surftest SJ-500 contact profilometer (Mitutoyo, Tokio, Japan) in compliance with the ISO 21920 standard [17], applying the sampling length λc = lr = 0.25 mm and the evaluation length ln = 1.25 mm (Figure 1). For each specimen, 4 measurements of roughness were made at randomly selected places.

**Figure 2:**
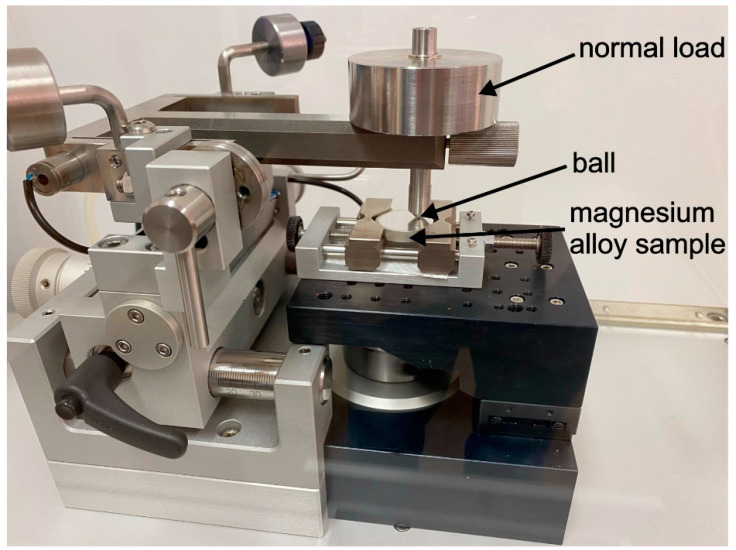
Schematic representation of the experimental set prepared for the tribological test.

Tribological tests were carried out on a TRN tribometer (Anton Paar, Corcelles-Cormondrèche, Switzerland) in a ball-on-flat system in linear reciprocating motion (Figure 2). For each type of sample, 2 sets consisting of 4 wear tracks were made. AISI 316L, ZrO_2_, Si_3_N_4_, and WC balls with a diameter of ∅ = 3 mm were used as counter-specimens. The basic properties of the balls according to the manufacturer’s certification and the calculated maximum Hertzian stress values according to our own analyses for the tribological association with the magnesium alloys WE43 and WE54 are provided in Table 3.

The wear was examined under technically dry friction conditions at room temperature of 21 ± 1 °C and humidity of 40 ± 5%, in accordance with the recommendations of the VAMAS Technical Note, ASTM G99, and ASTM G133 standards [12,18,19]. During tribological tests, the following parameters were determined: average area of the wear track P, specific wear rate k, and mean friction coefficient µ. Table 4 presents the tribological test parameters (the parameters were selected based on the superficial hardness and other sample conditions).

**Figure 3:**
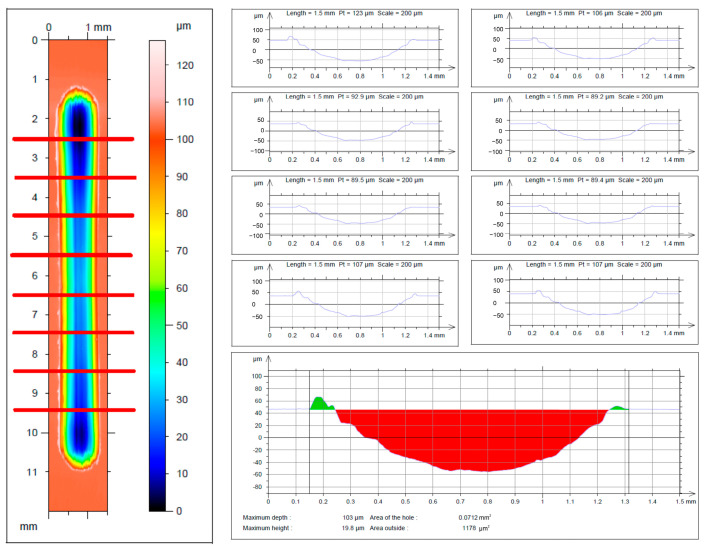
Procedure for measuring the average wear track area in linear reciprocating motion according to modified ASTM G133 and ASTM D7755 standards [12,13].

The mean surface area of the wear track area P was determined using the Form Talysurf Series 2-50i profilometer (Taylor-Hobson, Leicester, UK) according to modified ASTM G133 and ASTM D7755 standards [12,13]. This modification involved extending the number of measured profiles from 4 suggested by the standard to 8, allowing for a significant reduction in measurement error compared to the standard procedure (Figure 3). The results were processed using TalyMap Universal software (version 3.2.0) and OriginPro (version 2024; OriginLab Corporation, Northampton, MA, USA). The wear track formed during tribological tests has also been illustrated by acquiring an isometric 3D image of the surface studied along with a color change result map. The entire area of the wear track, i.e., 17 mm × 14 mm, was examined while maintaining the sampling distance of x = 1 μm and y = 25 μm. Images of 16 mm × 6 mm cross-sections were subsequently prepared.

The specific wear rate k was subsequently determined from the following formula [20,21]:

$$k=\frac{V}{L\cdot D}\left[\frac{{mm}^{3}}{N\cdot m}\right]$$

where L—the load applied (5 N), D—friction distance (50 m), and V—volume of the wear track (mm^3^).

Statistical analysis was performed using one-way ANOVA, followed by Tukey’s test using OriginPro version 2024 software (OriginLab Corporation, Northampton, MA, USA).

The morphology of the wear tracks and wear products was conducted using a JEOL JSM-6480 scanning electron microscope (Jeol, Tokyo, Japan); magnification from 30× to 2000× equipped with an adapter for X-ray microanalysis by the EDS method (IXRF, Austin, TX, USA); accelerating voltage ≤ 20 keV; takeoff angle of 35.0°; and elapsed lifetime of 30.0.

The phase composition of the material was analyzed using X-ray diffraction (XRD) with a Philips X’Pert PW 3040/60 diffractometer (Philips PANalytical in Almelo, The Netherlands). The equipment utilized a copper anode tube (CuKα wavelength of 1.54178 Å) operated at a 30 mA current and 40 kV. A graphite monochromator on the diffracted beam was employed to select the wavelength emitted by the copper anode. The diffractometer was set to operate in “step scanning” mode with a 0.04° step size and a 25-s counting time per step, covering an angular range from 10° to 140° 2θ. The setup included a 1/2° divergence slit for both the incident and diffracted beams, with 2° Soller slits also being utilized. To interpret the obtained diffractograms, the ICDD Card PDF 4 database was consulted.

## 3. Research Results and Discussion

Magnesium alloys with rare earth metals with different yttrium contents in the composition of WE43 (4 wt.%) and WE54 (5.2 wt.%) were selected for tests in reciprocating motion. Yttrium (Y) in combination with other rare earth metals (RE) acts as an alloying component, decreasing the grain size, increasing tensile strength at elevated temperatures, and increasing creep resistance [22]. Changes in the structure of WE43 and WE54 alloys obtained by applying a complex heat treatment consisting of solution treatment, aging, and cryogenic treatment in liquid nitrogen (before and after aging) resulted in improved micromechanical and mechanical properties such as an increase in H_IT_ hardness, E_IT_ Young’s modulus, and compressive strength—UCS (R_c_). These studies have been described in more detail in articles [15,16]. The improvement of these properties, in turn, had a direct impact on the tribological wear tested in four different material couples.

**Figure 4:**
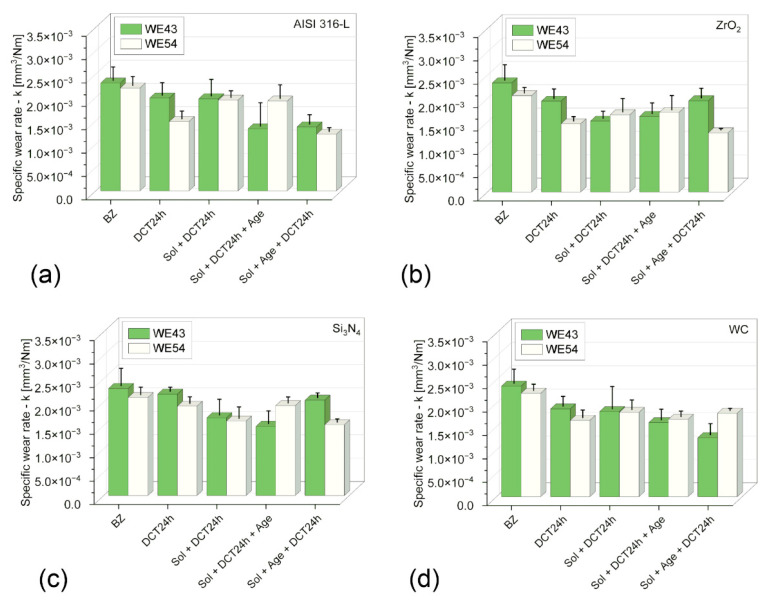
Specific wear rate k variations—of WE43 and WE54 magnesium alloys in material couples tested AISI 316-L—(**a**); ZrO_2_—(**b**); Si_3_N_4_—(**c**); WC—(**d**); in the initial state and after a complex heat treatment process (precipitation hardening—sub-zero treatment).

**Figure 5:**
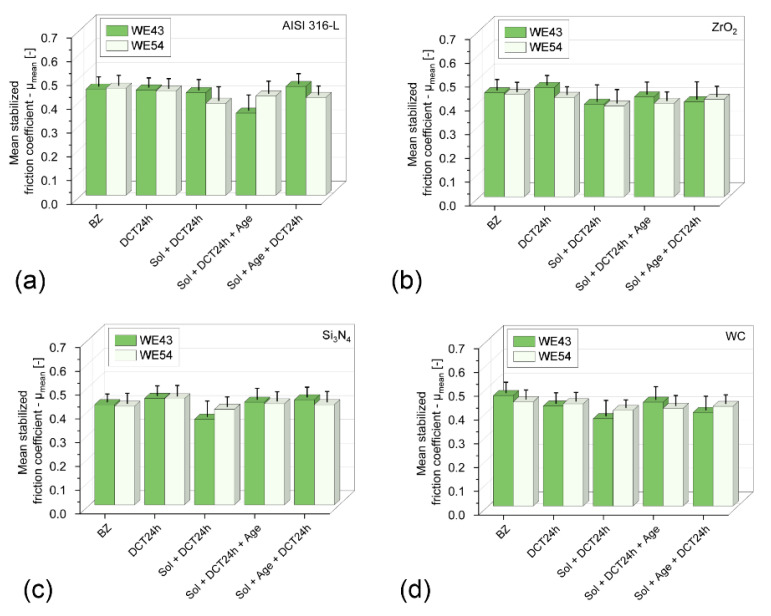
Mean stabilized friction coefficient µ_mean_ variations—of WE43 and WE54 magnesium alloys in material couples tested AISI 316-L—(**a**); ZrO_2_—(**b**); Si_3_N_4_—(**c**); WC—(**d**); in the initial state and after a complex heat treatment process (precipitation hardening—sub-zero treatment).

Table 5, Table 6, Table 7 and Table 8 and Figure 4 and Figure 5 provide detailed results of the tribological tests performed.

**Figure 6:**
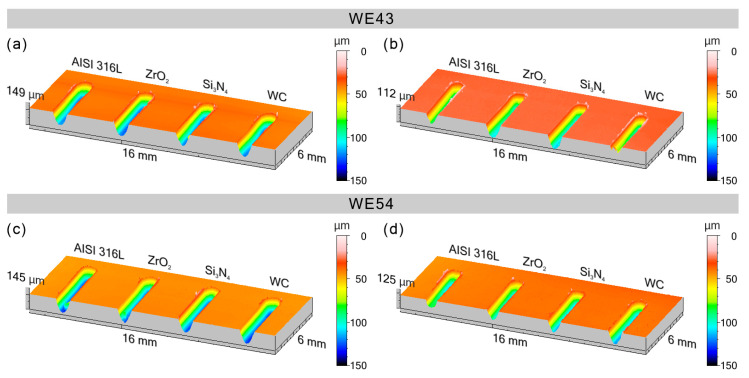
Three-dimensional isometric images of the cross-sections of the wear track formed as a result of linear reciprocating motion: WE43 alloy in the initial state—(**a**); WE43 alloy after sub-zero treatment before aging—(**b**); WE54 alloy—in the initial state—(**c**); WE54 alloy after sub-zero treatment before and after aging—(**d**).

Wear tests performed in linear reciprocating motion revealed that in each of the four material couples tested (AISI 316−L; ZrO_2_; Si_3_N_4_; WC) in the initial state, WE43 and WE54 magnesium alloys showed little resistance to sliding wear. The average volume of the wear track V (Table 5, Table 6, Table 7 and Table 8) in the initial state for WE43 alloy ranged from 0.572 to 0.591 [mm^3^], while for WE54 alloy it ranged from 0.516 to 0.553 [µm^2^]. Specific wear rate k for WE43 alloy ranged from 2.29 to 2.36∙10^−3^ [mm^3^/Nm] and 2.06 to 2.21∙10^−3^ [mm^3^/Nm] for WE54 (Table 5, Table 6, Table 7 and Table 8, Figure 4). The implementation of deep cryogenic treatment (DCT) and complex heat treatment in the form of precipitation hardening performed at an appropriately selected temperature combined with sub−zero treatment of both alloys allowed an approx. 20−45% reduction in specific wear rate for the WE43 alloy and an approx. 10−47% reduction in specific wear rate for the alloy. For WE43 alloy, the most advantageous results were obtained during friction against AISI 316−L steel for specimens subjected to solution treatment at 545 °C for 8 h, followed by sub−zero treatment at −196 °C for 24 h and aging at 225 °C for 24 h (V = 0.332 [mm^3^], k = 1.33∙10^−3^ [mm^3^/Nm]). The WE54 alloy also displayed the best tribological properties when working with a ball made of AISI 316−L steel, and similar results were also obtained using ZrO_2_ balls as a counter specimen. Such results were obtained after supersaturation at 545 °C for 8 h, sub−zero treatment in liquid nitrogen at −196 °C for 24 h, aging at 250 °C for 24 h, and again sub−zero treatment for 24 h (V = 0.304 [mm^3^], k = 1.22∙10^−3^ [mm^3^/Nm]) (Table 5, Table 6, Table 7 and Table 8, Figure 4). In comparison with an alloy with a lower yttrium content in the composition (WE43), the number of individual steps of the complex heat treatment and the aging temperature are changed for the most favorable tribological properties. During friction in linear reciprocating motion, WE54 alloy has more advantageous tribological properties after sub-zero treatment applied before and after aging, whereas WE43 alloy has more advantageous tribological properties after deep cryogenic treatment performed before aging. The findings above are also supported by the exemplary 3D isometric images of the cross-sections of the wear track presented in Figure 6, in which a significant reduction in the area of the wear marks can be observed for the suggested complex heat treatment.

The improvement of tribological properties as a result of the proposed complex heat treatment is mainly related to the modification of the microstructure of the alloys—the formation of a much larger amount of β’ phase precipitates—Mg_46.1_Y_6_._25_RE_3.45_ and the presence of other phases such as Mg_41_Nd_5_, La_0.5_RE, Mg_3_RE, Mg_2_Nd, MgRE, MgY, and Y_0.65_RE_0.35_ in the alloy subjected to sub-zero treatment in combination with additional heat treatment. These lamellar precipitates modify the mechanical, micromechanical, and sclerometric properties of the alloy [16,23], leading to an improvement in the service life of both alloys, which is directly evident through a significant improvement in tribological properties and a reduction in wear. The solution treatment of WE43 and WE54 alloys results in the dissolution of intermetallic phases in the solid solution matrix and an increase in the average grain area of the α-Mg solid solution [22]. Similar observations can be found in the relevant literature [24]. Mg-RE alloys are characterized by excellent aging properties due to the higher solubility of the Mg matrix [1,22]. In the process of aging, the supersaturated solid magnesium solution decays according to the scheme: α-Mg → β” → β’→ β_1_ → β [22,25,26,27]. The most advantageous results are obtained if significant amounts of precipitates consisting of a finely dispersed β’ phase appear in the structure [25]. Implementing a complex heat treatment in the form of a combination of precipitation hardening and deep cryogenic treatment considerably promotes this process, as shown in previous work on both alloys [14,15,16], which has a direct impact on tribological properties regardless of the material couple used. Wear on the balls (counter-specimens made of AISI316-L, ZrO_2_, Si_3_N_4_, and WC) was not recorded, among other reasons, due to the large difference in hardness of the materials tested compared to WE43 and WE54 magnesium alloys. The decrease in tribological wear was also influenced by a reduction in the friction coefficient. The mean stabilized friction coefficient μ_mean_ for both alloys tested decreased by approximately 10 to 20% depending on the heat treatment applied and the material couple (Figure 5). The most advantageous results, as in the case of specific wear rate, were recorded for the magnesium alloy-steel AISI 316-L couple. It is noteworthy that specimens subjected to solution treatment alone also display a low friction coefficient; this is related to a significant reduction in the hardness of the alloy after the first stage of the complex heat treatment. This relationship was recorded for all the material couples examined.

**Figure 7:**
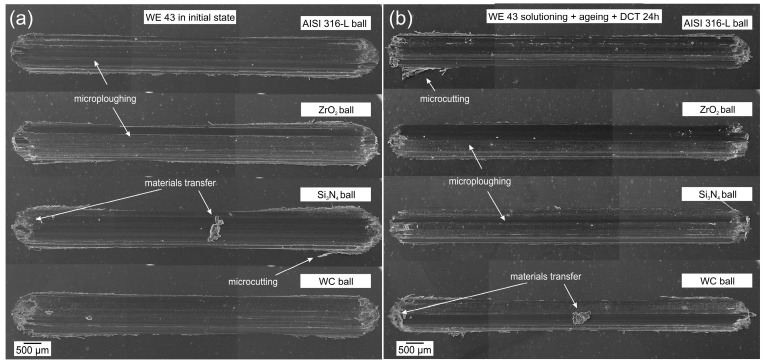
Morphology of WE43 magnesium alloy wear tracks formed in the reciprocating motion of the tested material couples in the initial state (**a**) and after the complex heat treatment process (precipitation hardening—sub-zero treatment) (**b**).

**Figure 8:**
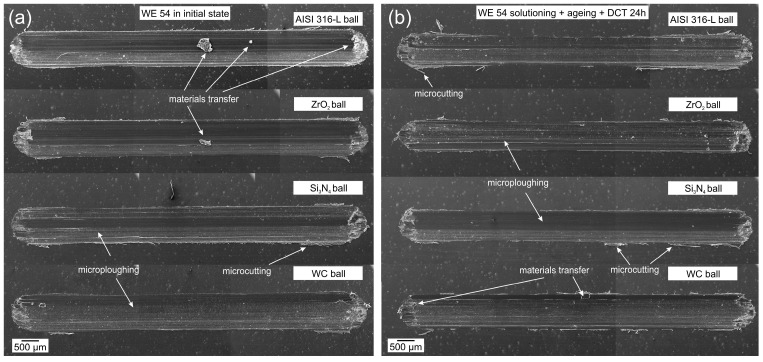
Morphology of WE54 magnesium alloy wear tracks formed in the reciprocating motion of the tested material couples in the initial state (**a**) and after the complex heat treatment process (precipitation hardening—sub-zero treatment) (**b**).

Tests of the morphology of the wear tracks (Figure 7 and Figure 8) and the morphology of wear products (Figure 7) have provided information on the wear mechanisms and types of wear products formed during dry friction of WE43 and WE54 alloys. Figure 5 illustrates exemplary SEM images of WE43 magnesium alloy wear tracks after tribological testing during dry friction in linear reciprocating motion for all material couples (Figure 7a), after precipitation treatment combined with deep cryogenic treatment (Figure 7b), and in the Figure 8 images after tribological interaction of WE54 magnesium alloy.

Following the analysis of tribological tests (specific wear rate of the order of 10^−3^ mm^3^/Nm) performed in reciprocating motion of a linear nature, a detailed evaluation of the wear mechanisms was carried out for the two magnesium alloys with rare earth metals tested (WE43 and WE54). Abrasive wear has been identified as the dominant mechanism of surface degradation in both cases. It is characterized by such failure mechanisms as the formation of numerous grooves and polishing that are arranged parallel to the direction of sliding motion [20,21]. These observations are consistent with generally accepted tribological models describing interactions between surfaces in relative motion. Moreover, areas of microploughing and microcutting, as well as adhesion, were identified on the friction surfaces, which were particularly pronounced at the extremities of the wear tracks. It should be mentioned that these areas formed when the direction of motion changed, which indicates that the dynamics of the tribological process is complex and that the interactions between the surfaces are closely related to the direction of the frictional forces.

**Figure 9:**
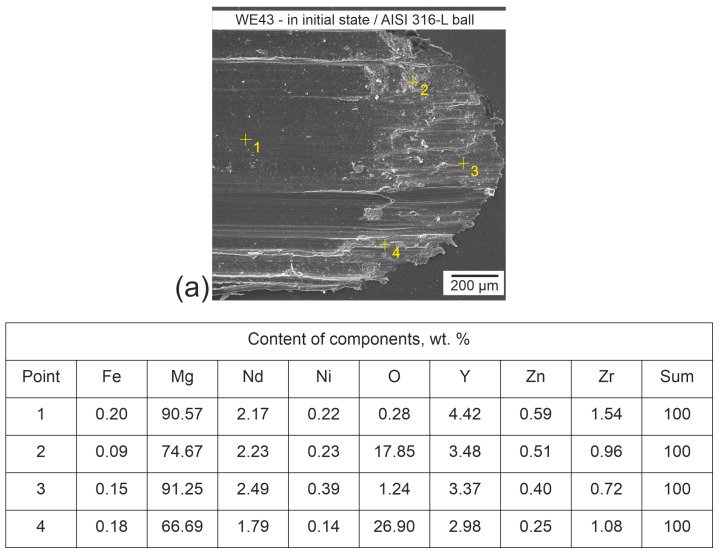

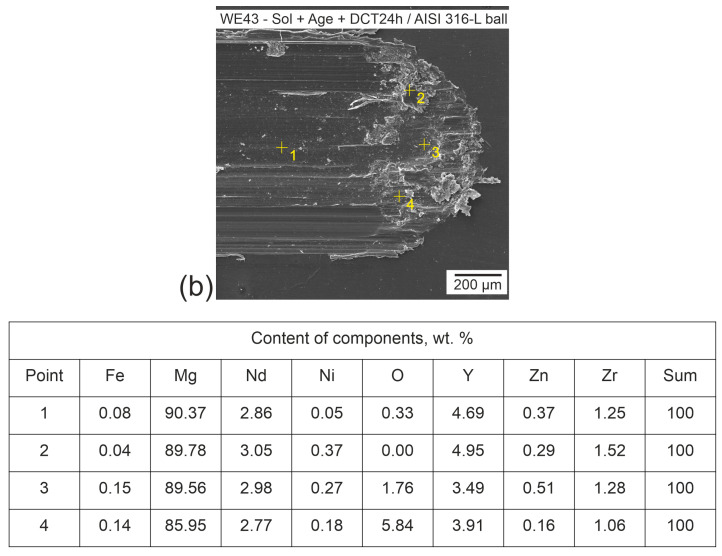
Microanalysis of the chemical composition (EDS) of WE43 alloy wear tracks in the as-delivered condition (**a**) and after DCT combined with precipitation hardening (**b**) after a tribological test with an AISI 316-L ball.

Example results of microanalysis of the chemical composition (EDS) of the wear tracks formed during the change of direction of motion after tribological cooperation with an AISI 316L ball in the initial state and after complex heat treatment are shown in Figure 9. All mechanisms indicative of abrasive wear can be observed, as well as areas of adhesion during the change of direction of motion. However, no deposition of wear products of the counter-partner—the ball—was observed, which confirms the absence of wear observed during profilometric and microscopic measurements. In addition, the results of the analysis are consistent with the alloy certificate provided by the manufacturer, Luxfer Mel Technologies (Manchester, UK).

In order to reduce wear processes, deep cryogenic treatment was introduced in addition to the classic heat treatment in the form of precipitation hardening (solution treatment and aging). The effect of the complex heat treatment was a significant reduction in the severity of tribological wear, which was confirmed by a comparative analysis of the wear tracks in the initial state and after applying different variants of heat treatment. The wear tracks were considerably reduced, which demonstrates the high effectiveness of the applied method in terms of improving the tribological properties of the tested materials for each of the tested material couples (Figure 7b, Figure 8b and Figure 9b). The findings of the study emphasize the potential for the application of deep cryogenic treatment as an innovative method for manufacturing components for which high tribological wear resistance is required. This study opens up new perspectives for further research into the optimization of treatment processes in order to obtain materials with even better operational properties.

**Figure 10:**
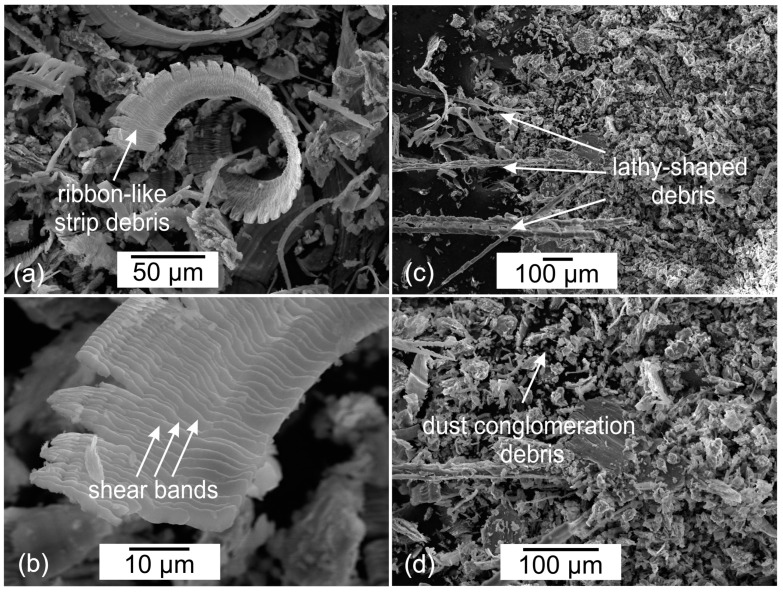
Morphology of wear products of magnesium alloys WE43 and WE54 formed in linear reciprocating motion: ribbon-like strip debris (**a**,**b**); lathy-shaped debris (**c**); dust conglomeration debris (**d**).

In order to gain a better insight into and understanding of the wear mechanisms of WE43 and WE54 magnesium alloys, a study of the morphology of the wear tracks was also carried out. Figure 10a,b show typical ribbon-like strip debris with machining characteristics. The ribbon-like strip is smooth on one side and serrated on the opposite side. This indicates an abrasive micro-cutting mechanism. Figure 10b further provides a close-up image in which parallel stripes become visible, indicating the formation of shear bands. These observations are consistent with those reported in the literature [28,29]. Figure 10c displays lathy-shaped debris, which is also indicative of a sliding wear mechanism, but in this case the debris particles have been separated from the surface of the magnesium alloy due to friction and have fractured. In Figure 10d, on the other hand, wear products in the form of dust can be observed (this may indicate an oxidation effect, which is not the dominant wear mechanism in this case). The dust conglomeration debris in Figure 10d and associated morphologies shown in Figure 7, Figure 8 and Figure 9 to reduce wear processes can be correlated with a similar morphology described in the article [30]. The deep cryogenic treatment introduced into the precipitation hardening process of the studied magnesium alloys by reducing wear also reduces the formation of wear products, the occurrence of which in the friction node has a detrimental effect on tribological properties.

## 4. Summary and Conclusions

This paper reports tribological tests on WE43 and WE54 magnesium alloys performed in linear reciprocating motion for four different material couples (AISI 316-L, ZrO_2_, Si3N_4_, and WC). Additionally, magnesium alloys were subjected to a complex heat treatment in the form of solution treatment, sub-zero treatment, and aging in order to investigate the proposed treatment in the context of tribological interaction at different friction nodes. For each of the four material couples, 10 different specimen variants were tested (40 variants in total). Wear tests in a reciprocating motion were performed alongside friction coefficient measurements, profilometric studies before and after friction, and morphology tests of the wear tracks and wear products. An analysis of the research results allows the formulation of the following conclusions:

1. The complex heat treatment resulted in the formation of significant amounts of β’ and other phase precipitates, which, through changes in microstructure and mechanical properties, also affected the tribological test results obtained.
2. Precipitation treatment combined with deep cryogenic treatment reduces the specific wear rate for WE43 and WE54 by 20–45% and 10–47%, respectively, depending on the material couple used, demonstrating the high effectiveness of the proposed complex heat treatment in terms of improving the tribological properties of these materials. The most advantageous results were obtained during friction against AISI 316-L steel for both WE43 and WE54 (an almost twofold reduction in the average volume of the wear track and specific wear rate). Favorable wear results were also obtained for the material coupled with ZrO_2_ (especially for the WE54 alloy). A reduction in the mean stabilized friction coefficient of approximately 10–20% was also observed, depending on the material couple and complex heat treatment applied.
3. In order to obtain the most advantageous tribological properties, the number of individual stages of the complex heat treatment and the aging temperature are modified. WE54 alloy has more advantageous tribological properties after sub-zero treatment applied before and after aging, whereas WE43 alloy has more advantageous tribological properties after deep cryogenic treatment performed before aging.
4. Studies of the morphology of the wear tracks revealed that the predominant wear mechanism was abrasion, characterized by the formation of grooves and polishing on the friction surface; wear mechanisms such as microploughing, microcutting, and adhesion were present.
5. Morphology analysis of the wear tracks further revealed the formation of several types of wear products: ribbon-like strip debris, lathy-shaped debris, and dust conglomeration debris, which also testify to the abrasive nature of the wear process of WE43 and WE54 alloys in linear reciprocating motion. Deep cryogenic treatment combined with precipitation hardening effectively reduces the amount of wear products formed.
6. The results of the study demonstrate the effectiveness of complex heat treatment, including deep cryogenic treatment, as a method to improve the tribological wear resistance of magnesium alloys with rare earth metals. This offers the potential for new applications requiring high tribological wear resistance.
7. During profilometry measurements of wear track areas, the ASTM G133 and ASTM D7755 standards were modified by extending the number of measured profiles from four to eight. This modification allows for a significant reduction in measurement error compared to the standard measurement procedure.

## Figures and Tables

**Table 1:** Certified chemical composition of WE43 and WE54 alloys in the as-delivered condition.

Component Content, wt.%						
Alloy Type	Y	RE	Zr	Zn	Cu	Mn
WE43	4.0	5.3	0.49	0.01	0.002	0.02
WE54	5.2	4.3	0.5	0.01	0.002	0.009

**Table 2:** Heat treatment of WE43 and WE54 magnesium alloys.

Sample	Stage of Heat Treatment		
	Solution Treatment (Sol)	Deep Cryogenic Treatment (DCT 24 h)	Aging (Age)
WE43—BZ	-	-	-
WE54—BZ	-	-	-
WE43—DCT 24 h	-	−196 °C/24 h	-
WE54—DCT 24 h	-	−196 °C/24 h	-
WE43—Sol + DCT 24 h	545 °C/8 h	−196 °C/24 h	-
WE54—Sol + DCT 24 h	545 °C/8 h	−196 °C/24 h	-
WE43—Sol + DCT 24 h + Age	545 °C/8 h	−196 °C/24 h	225 °C/24 h
WE54—Sol + DCT 24 h + Age	545 °C/8 h	−196 °C/24 h	250 °C/24 h
WE43—Sol + Age + DCT 24 h	545 °C/8 h	−196 °C/24 h (before and after aging)	225 °C/24 h
WE54—Sol + Age + DCT 24 h	545 °C/8 h	−196 °C/24 h (before and after aging)	250 °C/24 h

**Table 3:** Selected properties of balls used in tribological tests along with analysis of maximum Hertzian stress at individual contact points.

Property	Symbol and Unit	Values			
		AISI 316-L	ZrO_2_	Si_3_N_4_	WC
Density	δ [g/cm³]	7.95	6.0	3.26	14.95
Young’s modulus	E [GPa]	200	213	300	650
Specific heat	c [J/kg∙K]	500	450	740	225
Coefficient of linear thermal expansion	A [10^−6^/°C]	17	9.8	3.4	5.9
Thermal conductivity	λ [W/(m∙K)]	15.0	3.3	25.0	87.0
Hardness	-	10–25 HRC	87–91 HRA	1400–1600 HV	1550–1780 HV
Ultimate tensile strength	UTS [MPa]	550–1250	-	-	-
Ultimate compressive strength	UTS [MPa]	-	1750–2500	2300–4000	5500–5800
Poisson ratio	ν [-]	0.30	0.32	0.28	0.20
Maximum Hertzian stress with magnesium alloy (WE43, WE54) tribological couple—own analysis	(*σ_c_*)_*max*_ [MPa]	888.2	896.3	925.4	970.2

**Table 4:** Tribological test parameters.

Linear Reciprocating Motion	
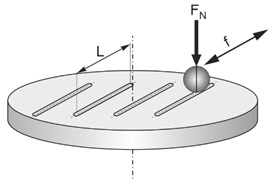	
Load L	5 N
Amplitude s	10 mm
Frequency f	2.5 Hz
Friction distance D	50 m
Max. linear velocity v	7.85 cm/s

**Table 5:** Results of tribological tests on a friction couple WE43 and WE54 magnesium alloy—AISI316L in linear reciprocating motion.

Name of Specimen	Average Volume of the Wear Track V [mm^3^]		Specific Wear Rate k∙10^−3^ [mm^3^/Nm]		Mean Stabilized Friction Coefficient µ_mean_ [-]	
	Value	Standard Deviation	Value	Standard Deviation	Value	Standard Deviation
WE43—BZ	0.577	0.073	2.31	0.29	0.448	0.040
WE54—BZ	0.550	0.048	2.20	0.19	0.451	0.043
WE43—DCT 24 h	0.497	0.069	1.99	0.28	0.443	0.040
WE54—DCT 24 h	0.372	0.042	1.49	0.17	0.439	0.041
WE43—Sol + DCT 24 h	0.494	0.090	1.97	0.36	0.432	0.045
WE54—Sol + DCT 24 h	0.486	0.036	1.95	0.15	0.387	0.058
WE43—Sol + DCT 24 h + Age	0.332	0.126	1.33	0.51	0.346	0.066
WE54—Sol + DCT 24 h + Age	0.481	0.073	1.92	0.29	0.418	0.051
WE43—Sol + Age + DCT 24 h	0.343	0.052	1.37	0.21	0.458	0.043
WE54—Sol + Age + DCT 24 h	0.304	0.064	1.22	0.26	0.412	0.037

Statistically significant results, where *p*-value < 0.05; one-way ANOVA followed by Tukey’s test.

**Table 6:** Results of tribological tests on a friction couple WE43 and WE54 magnesium alloy—ZrO_2_ in linear reciprocating motion.

Name of Specimen	Average Volume of the Wear Track V [mm^3^]		Specific Wear Rate k∙10^−3^ [mm^3^/Nm]		Mean Stabilized Coefficient of Dry Friction µ_mean_ [-]	
	Value	Standard Deviation	Value	Standard Deviation	Value	Standard Deviation
WE43—BZ	0.585	0.084	2.34	0.34	0.438	0.045
WE54—BZ	0.516	0.030	2.06	0.12	0.431	0.040
WE43—DCT 24 h	0.486	0.052	1.94	0.21	0.460	0.040
WE54—DCT 24 h	0.366	0.024	1.46	0.10	0.419	0.034
WE43—Sol + DCT 24 h	0.380	0.039	1.52	0.16	0.390	0.070
WE54—Sol + DCT 24 h	0.414	0.074	1.66	0.30	0.383	0.058
WE43—Sol + DCT 24 h + Age	0.405	0.059	1.62	0.23	0.421	0.052
WE54—Sol + DCT 24 h + Age	0.426	0.077	1.70	0.31	0.394	0.037
WE43—Sol + Age + DCT 24 h	0.489	0.053	1.96	0.21	0.400	0.073
WE54—Sol + Age + DCT 24 h	0.318	0.050	1.27	0.20	0.410	0.044

Statistically significant results, where *p*-value < 0.05; one-way ANOVA followed by Tukey’s test.

**Table 7:** Results of tribological tests on a friction couple WE43 and WE54—Si_3_N_4_ in linear reciprocating motion.

Name of Specimen	Average Volume of the Wear Track V [mm^3^]		Specific Wear Rate k∙10^−3^ [mm^3^/Nm]		Mean Stabilized Coefficient of Dry Friction µ_mean_ [-]	
	Value	Standard Deviation	Value	Standard Deviation	Value	Standard Deviation
WE43—BZ	0.572	0.094	2.29	0.38	0.422	0.034
WE54—BZ	0.524	0.042	2.10	0.17	0.415	0.043
WE43—DCT 24 h	0.542	0.024	2.17	0.09	0.448	0.042
WE54—DCT 24 h	0.478	0.036	1.91	0.15	0.447	0.045
WE43—Sol + DCT 24 h	0.418	0.085	1.66	0.34	0.360	0.066
WE54—Sol + DCT 24 h	0.398	0.062	1.59	0.25	0.402	0.041
WE43—Sol + DCT 24 h + Age	0.370	0.069	1.48	0.28	0.432	0.048
WE54—Sol + DCT 24 h + Age	0.480	0.033	1.92	0.13	0.426	0.040
WE43—Sol + Age + DCT 24 h	0.510	0.025	2.04	0.10	0.443	0.042
WE54—Sol + Age + DCT 24 h	0.378	0.057	1.51	0.23	0.422	0.045

Statistically significant results, where *p*-value < 0.05; one-way ANOVA followed by Tukey’s test.

**Table 8:** Results of tribological tests on a friction couple WE43 and WE54 magnesium alloy—WC in linear reciprocating motion.

Name of Specimen	Average Volume of the Wear track V [mm^3^]		Specific Wear Rate k∙10^−3^ [mm^3^/Nm]		Mean Stabilized Coefficient of Dry Friction µ_mean_ [-]	
	Value	Standard Deviation	Value	Standard Deviation	Value	Standard Deviation
WE43—BZ	0.591	0.077	2.36	0.31	0.465	0.045
WE54—BZ	0.553	0.035	2.21	0.14	0.438	0.039
WE43—DCT 24 h	0.469	0.053	1.88	0.21	0.421	0.045
WE54—DCT 24 h	0.408	0.042	1.63	0.17	0.430	0.037
WE43—Sol + DCT 24 h	0.456	0.012	1.82	0.48	0.368	0.066
WE54—Sol + DCT 24 h	0.451	0.053	1.80	0.21	0.403	0.033
WE43—Sol + DCT 24 h + Age	0.396	0.058	1.59	0.23	0.437	0.054
WE54—Sol + DCT 24 h + Age	0.414	0.030	1.66	0.12	0.411	0.043
WE43—Sol + Age + DCT 24 h	0.316	0.061	1.26	0.24	0.394	0.058
WE54—Sol + Age + DCT 24 h	0.446	0.019	1.78	0.08	0.420	0.038

Statistically significant results, where *p*-value < 0.05; one-way ANOVA followed by Tukey’s test.

## Data Availability

The data presented in this study are available on request from the corresponding author. The data are not publicly available due to privacy reasons.

## References

[1] Rokhlin L.L. (2003). Magnesium Alloys Containing Rare Earth Metals.

[2] Satya Prasad S.V., Prasad S.B., Verma K., Kumar Mishra R., Kumar V., Singh S. (2022). The Role and Significance of Magnesium in Modern Day Research-A Review. J. Magnes. Alloys.

[3] Antoniac I., Miculescu M., Manescu V., Stere A., Quan P.H., Paltânea G., Robu A., Earar K. (2022). Magnesium-Based Alloys Used in Orthopedic Surgery. Materials.

[4] Witte F. (2010). The History of Biodegradable Magnesium Implants: A Review. Acta Biomater..

[5] Kose O. Magnesium (MgYREZr) Bioabsorbable Screws in Orthopedic Surgery. https://military-medicine.com/article/3830-magnesium-mgyrezr-bioabsorbable-screws-in-orthopedic-surgery.html.

[6] Staiger M.P., Pietak A.M., Huadmai J., Dias G. (2006). Magnesium and Its Alloys as Orthopedic Biomaterials: A Review. Biomaterials.

[7] Waizy H., Diekmann J., Weizbauer A., Reifenrath J., Bartsch I., Neubert V., Schavan R., Windhagen H. (2014). In Vivo Study of a Biodegradable Orthopedic Screw (MgYREZr-Alloy) in a Rabbit Model for up to 12 Months. J. Biomater. Appl..

[8] Rahim M.I., Eifler R., Rais B., Mueller P.P. (2015). Alkalization Is Responsible for Antibacterial Effects of Corroding Magnesium. J. Biomed. Mater. Res..

[9] Kang Y.H., Huang Z.H., Wang S.C., Yan H., Chen R.S., Huang J.C. (2020). Effect of Pre-Deformation on Microstructure and Mechanical Properties of WE43 Magnesium Alloy II: Aging at 250 and 300 °C. J. Magnes. Alloys.

[10] Jin W., Wu G., Feng H., Wang W., Zhang X., Chu P.K. (2015). Improvement of Corrosion Resistance and Biocompatibility of Rare-Earth WE43 Magnesium Alloy by Neodymium Self-Ion Implantation. Corros. Sci..

[11] Ayerdi J.J., Aginagalde A., Llavori I., Bonse J., Spaltmann D., Zabala A. (2021). Ball-on-Flat Linear Reciprocating Tests: Critical Assessment of Wear Volume Determination Methods and Suggested Improvements for ASTM D7755 Standard. Wear.

[12] (2022). Standard Test Method for Linearly Reciprocating Ball-on-Flat Sliding Wear.

[13] (2022). Standard Practice for Determining the Wear Volume on Standard Test Pieces Used by High-Frequency, Linear-Oscillation (SRV) Test Machine.

[14] Barylski A., Aniołek K., Dercz G., Kupka M., Kaptacz S. (2021). The Effect of Deep Cryogenic Treatment and Precipitation Hardening on the Structure, Micromechanical Properties and Wear of the Mg-Y-Nd-Zr Alloy. Wear.

[15] Barylski A., Aniołek K. (2022). Effect of Deep Cryogenic Treatment Time on Micromechanical and Tribological Properties of Magnesium Alloys WE43 and WE54. Tribologia.

[16] Barylski A., Aniołek K., Dercz G., Matuła I., Rak J., Mazur I. (2023). The Effect of Changes in the Aging Temperature Combined with Deep Cryogenic Treatment on the Structure, Phase Composition, and Micromechanical Properties of the WE43 Magnesium Alloy. Materials.

[17] (2021). Geometrical Product Specifications (GPS). Surface Texture: Profile. Part 2: Terms, Definitions and Surface Texture Parameters..

[18] (2023). Standard Test Method for Wear Testing with a Pin-on-Disk Apparatus.

[19] Czichos H., Becker S., Lexow J. (1987). Multilaboratory Tribotesting: Results from the VAMAS Program on Wear Test Methods. Wear.

[20] García-León R.A., Martínez-Trinidad J., Zepeda-Bautista R., Campos-Silva I., Guevara-Morales A., Martínez-Londoño J., Barbosa-Saldaña J. (2021). Dry sliding wear test on borided AISI 316L stainless steel under ball-on-flat configuration: A statistical analysis. Tribol. Int..

[21] García-León R.A., Martínez-Trinidad J., Campos-Silva I., Figueroa-López U., Guevara-Morales A. (2021). Wear maps of borided AISI 316L steel under ball-on-flat dry sliding conditions. Mater. Lett..

[22] Oshida Y. (2021). Magnesium Materials: From Mountain Bikes to Degradable Bone Grafts.

[23] Barylski A., Aniołek K., Dercz G., Kupka M., Matuła I., Kaptacz S. (2021). The Sclerometrical, Mechanical and Wear Behavior of the Mg-Y-Nd Magnesium Alloy after Deep Cryogenic Treatment Combined with Heat Treatment. Materials.

[24] Kiełbus A. (2007). The Influence of Solution Treatment Time on the Microstructure of WE43 Magnesium Alloy. Acta Metall. Slovaca.

[25] Nie J.F., Muddle B.C. (2000). Characterisation of Strengthening Precipitate Phases in a Mg-Y-Nd Alloy. Acta Mater..

[26] Nie J.F. (2012). Precipitation and Hardening in Magnesium Alloys. Met. Mater. Trans. A.

[27] Kiełbus A. (2007). The Influence of Ageing on Structure and Mechanical Properties of WE54 Alloy. J. Achiev. Mater. Manuf. Eng..

[28] Samuels L.E., Doyle E.D., Turley D.M., Rigney D.A. (1981). Sliding Wear Mechanisms. Fundamentals of Friction and Wear of Materials.

[29] Campbell C.E., Bendersky L.A., Boettinger W., Ivester J.R. (2006). Microstructural characterization of Al-7075-T651 chips and work pieces produced by high-speed machining. Mater. Sci. Eng. A.

[30] Manickam M., Singh P., Issa T.B., Thurgate S., De Marco R. (2006). Lithium Insertion into Manganese Dioxide Electrode in MnO2/Zn Aqueous Battery: Part III. Electrochemical Behavior of γ-MnO_2_ in Aqueous Lithium Hydroxide Electrolyte. J. Power Sources.

